# The cognitive complexity of concurrent cognitive-motor tasks reveals age-related deficits in motor performance

**DOI:** 10.1038/s41598-018-24346-7

**Published:** 2018-04-17

**Authors:** Anderson Souza Oliveira, Mikkel Staall Reiche, Cristina Ioana Vinescu, Sif Amalie Halkjær Thisted, Carina Hedberg, Miguel Nobre Castro, Martin Gronbech Jørgensen

**Affiliations:** 1 Department of Materials and Production, Aalborg University, Aalborg, Denmark; 2 Department of Health Science and Technology, Aalborg University, Aalborg, Denmark; 3 Department of Geriatrics, Aalborg University Hospital, Aalborg, Denmark

## Abstract

Aging reduces cognitive functions, and such impairments have implications in mental and motor performance. Cognitive function has been recently linked to the risk of falls in older adults. Physical activities have been used to attenuate the declines in cognitive functions and reduce fall incidence, but little is known whether a physically active lifestyle can maintain physical performance under cognitively demanding conditions. The aim of this study was to verify whether physically active older adults present similar performance deficits during upper limb response time and precision stepping walking tasks when compared to younger adults. Both upper limb and walking tasks involved simple and complex cognitive demands through decision-making. For both tasks, decision-making was assessed by including a distracting factor to the execution. The results showed that older adults were substantially slower than younger individuals in the response time tasks involving decision-making. Similarly, older adults walked slower and extended the double support periods when precision stepping involved decision-making. These results suggest that physically active older adults present greater influence of cognitive demanding contexts to perform a motor task when compared to younger adults. These results underpin the need to develop interventions combining cognitive and motor contexts.

## Introduction

According to the Danish Health Authority between the years 2010–2012, approximately 75% of all emergency contacts to Danish hospitals made by adults aged +65 years were caused by fall accidents. From these, almost 29% were hospitalized and 10.5% subsequently died from the fall^1^. There are several risk factors associated with falls in older adults, such as: previous falls, polypharmacy, lower extremity weakness, inability to maintain a steady gait and other psychological or physical imbalances^2,3^. A growing body of researchers has been focusing on developing intervention programs directed towards fall-prone older adults^4,5^. Across the large variety of fall interventions, those focusing on vitamin supplementation, vision assessment/treatment, increased awareness of environmental hazards and physical activities have been proven somewhat effective^2,5^. However, the scientific community still seeks additional risk factors causing falls in older adults.

Humans can perform concurrent tasks such speaking and/or making calculations during walking, which combined are considered dual tasks. An extensive body of literature has shown that the performance of a motor task can be negatively influenced by a concurrent cognitive task across all lifespan^6–12^. These performance deficits can be primarily explained by the need to share brain resources between the concurrent motor and cognitive tasks^13^. With age, motor actions such as walking may require greater need of cognitive control and supervision, and this increased cognitive involvement on motor actions establishes an increased cost to accomplish the task^14,15^. It has been shown that performing combined cognitive and walking tasks require greater activation of the prefrontal cortex to cope with the dual-task context in young adults^8^. However, aging induces reduction in prefrontal activation and shifts of processing resources from this area to other brain areas when dual tasks are performed in older adults^16^. Ageing has been usually associated with reduced processing efficiency, which can diminish the capacity of some brain areas^17,18^ and impose a strong limitation to the execution of combined cognitive and motor tasks.

Approximately 10% of people aged +65 years suffer from some form of cognitive impairment, and this number increases to 50% in +85 years-old^19^. Ageing causes reduction in motor and cognitive performance, subsequently leading to increased response time, commonly defined as the capacity to respond to external stimuli or perturbations^3,20,21^. Based on such evidence, cognitive functions and processing speed are currently being considered as risk factors for falls^22–24^. The response time after a trip is highly relevant to avoid falls^20^, and the aged brain may present slower processing time in simple reaction time tasks^25^. Consequently, older adults present increased attentional demands to control posture and subsequently the risk of falls increases^17,26^. In addition, reduced executive functions in older adults directly influence postural control^11^, especially when a secondary task is present^10^. Therefore, it is highly relevant to further explore the performance of older adults in simple tasks, such as response time paradigms in different levels of postural demands to understand the limitations ageing imposes on motor performance.

Mobility and ambulation require special attention in the aged population, as the awareness to environmental hazards has been considered the most important cause for falls^27^. Particularly, the ability of older adults to overcome obstacles in their path is compromised with age^28,29^.Older adults present reduced walking speed and stride duration, as well as increased double support period, which are associated with reduced walking stability^17,30,31^. Moreover, older adults assume a more cautious walking pattern, which alters their ground reaction forces^32^. When stepping precision is required during walking, older adults presenting higher risk of falling present poorer performance than those at lower risks^33^. These deficits may occur due to deficits in the motor systems^17,30^, but may also be partially related to changes in the morphology of brain areas involved in modulating motor control and cognition^34^. These combined factors increase the difficulty to dissociate the causes of falls in older adults. Therefore, risk factors for falls in older adults are multi-factorial, and novel methods to explore factors related especially to cognition and processing speed during overground locomotion are needed^23^.

General physical activity has been successful in counteracting the decline in muscle strength^35^, postural control^26,36^, and cognitive performance caused by aging^19,37,38^. However, little is known whether physically active lifestyle can preserve performance in different types of combined cognitive and motor contexts, such as simple response time tasks and decision-making during walking. Moreover, if physically active older adults present poor performance in different types of cognitive-motor tasks, it is plausible that their activities are not sufficient to warrant appropriate cognitive stimuli to reduce fall risks. In this context, the aim of this study was to explore whether physically active older adults would present similar relative performance deficits compared to younger adults when cognitive complexity of a task is increased, during (1) a simple and complex response time task (upper extremity) while standing and walking on a treadmill, and (2) during a simple and complex precision stepping walking task. We therefore hypothesized that (1) the older group would experience greater declines in the performance of the response time and precision stepping tasks when these tasks involved higher cognitive complexity; (2) older adults would present proportional deficits in both response time and precision stepping task. In other words, older adults presenting greater influence of cognitive complexity on motor performance would display greater deficits in both response time and walking speed. Conversely, young adults would not present such behavior. The confirmation of these hypotheses can demonstrate that the motor performance of older adults may be globally influenced by declines in cognitive functions.

## Results

### Study population

**Figure 1:**
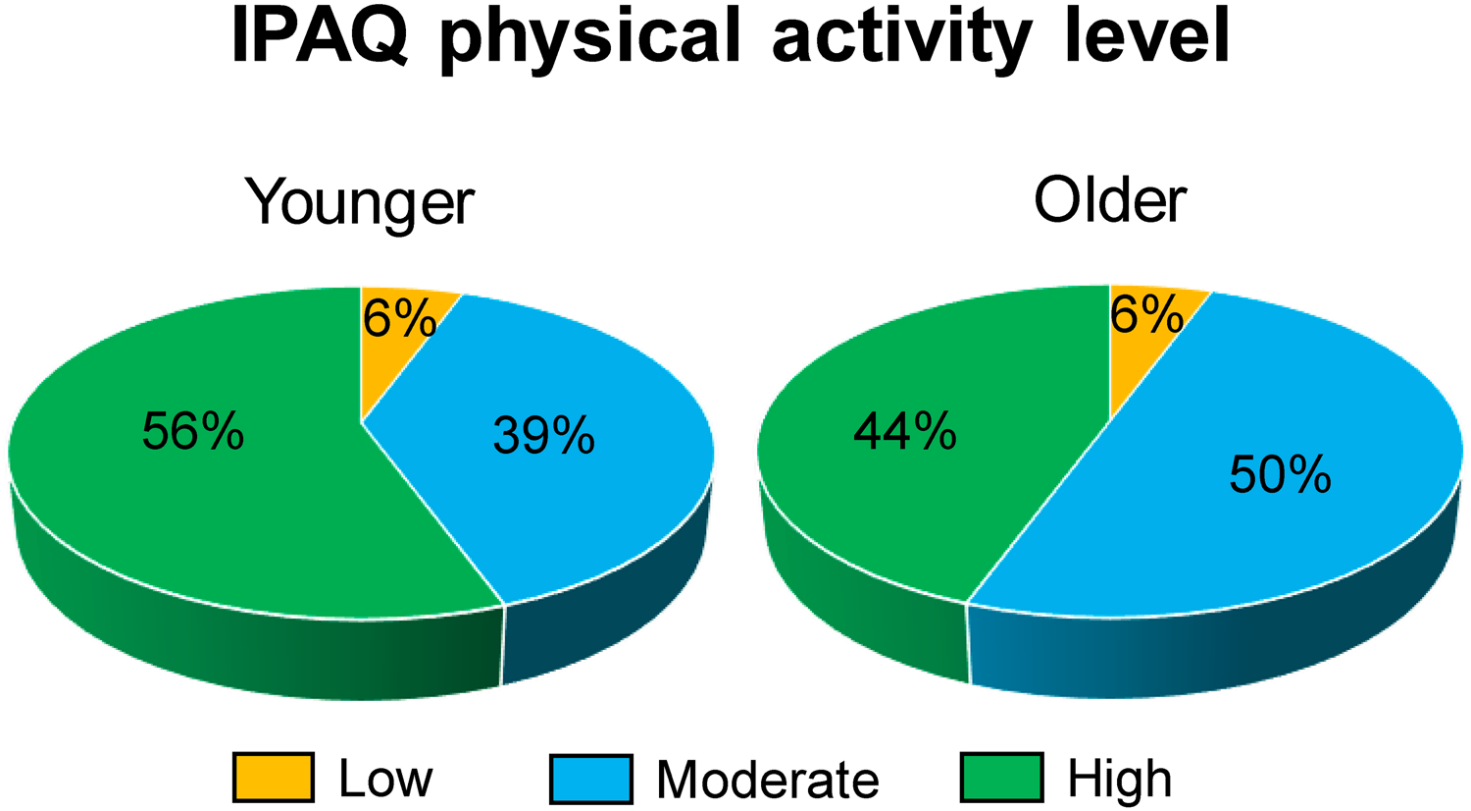
Distribution of participants with low, moderate and high level of physical activity divided by age.

There were no statistical differences in gender distribution, height, body mass and International Physical Activity Questionnaire (IPAQ) between the younger and older adults (p < 0.05, Table 1). The majority of participants in both groups presented moderate-to-high levels of physical activity (Fig. 1).

**Table 1:** Sample demographics, body mass index (BMI), International Physical Activity Questionnaire (IPAQ) score and amount mild, moderate and vigorous activities for younger and older adults.

	Younger	Older
Gender	10 F/8 M	10 F/8 M
Age (years)	24.22 ± 3.64	70.12 ± 4.90
Height (m)	1.75 ± 0.12	1.72 ± 0.08
Mass (kg)	74.47 ± 11.88	70.56 ± 15.23
BMI	24.06. ± 1.96	23.47 ± 3.19
IPAQ	2.5 ± 0.6	2.4 ± 0.6
Mild activities (min/week)	75 ± 50	28 ± 29
Moderate activities (min/week)	43 ± 24	76 ± 62
Vigorous activities (min/week)	34 ± 20	54 ± 37

### Postural sway during quiet stance

There were no significant main effects of limb (left *vs* right) or group (younger *vs* older) in the average center of pressure speed (p > 0.05, Table 2), as well as the variability of the center of pressure speed between younger and older adults (p > 0.05) in both the medial-lateral and anterior-posterior directions.

**Table 2:** Mean ± SD average center of pressure speed (SPEED) and center of pressure speed variability (VAR) in the medial-lateral (ML) and anterior-posterior (AP) directions for the younger and older adults.

	Left limb		Right limb	
	Yyounger	Older	Younger	Older
SPEED-ML (cm.s^−1^)	0.22 ± 0.08	0.20 ± 0.06	0.23 ± 0.05	0.22 ± 0.06
VAR-ML (cm.s^−1^)	0.19 ± 0.08	0.17 ± 0.05	0.19 ± 0.05	0.19 ± 0.06
SPEED-AP (cm.s^−1^)	0.97 ± 0.27	1.03 ± 0.32	1.02 ± 0.30	1.04 ± 0.31
VAR-AP (cm.s^−1^)	0.76 ± 0.21	0.89 ± 0.28	0.87 ± 0.37	0.86 ± 0.27

### Response time

**Figure 2:**
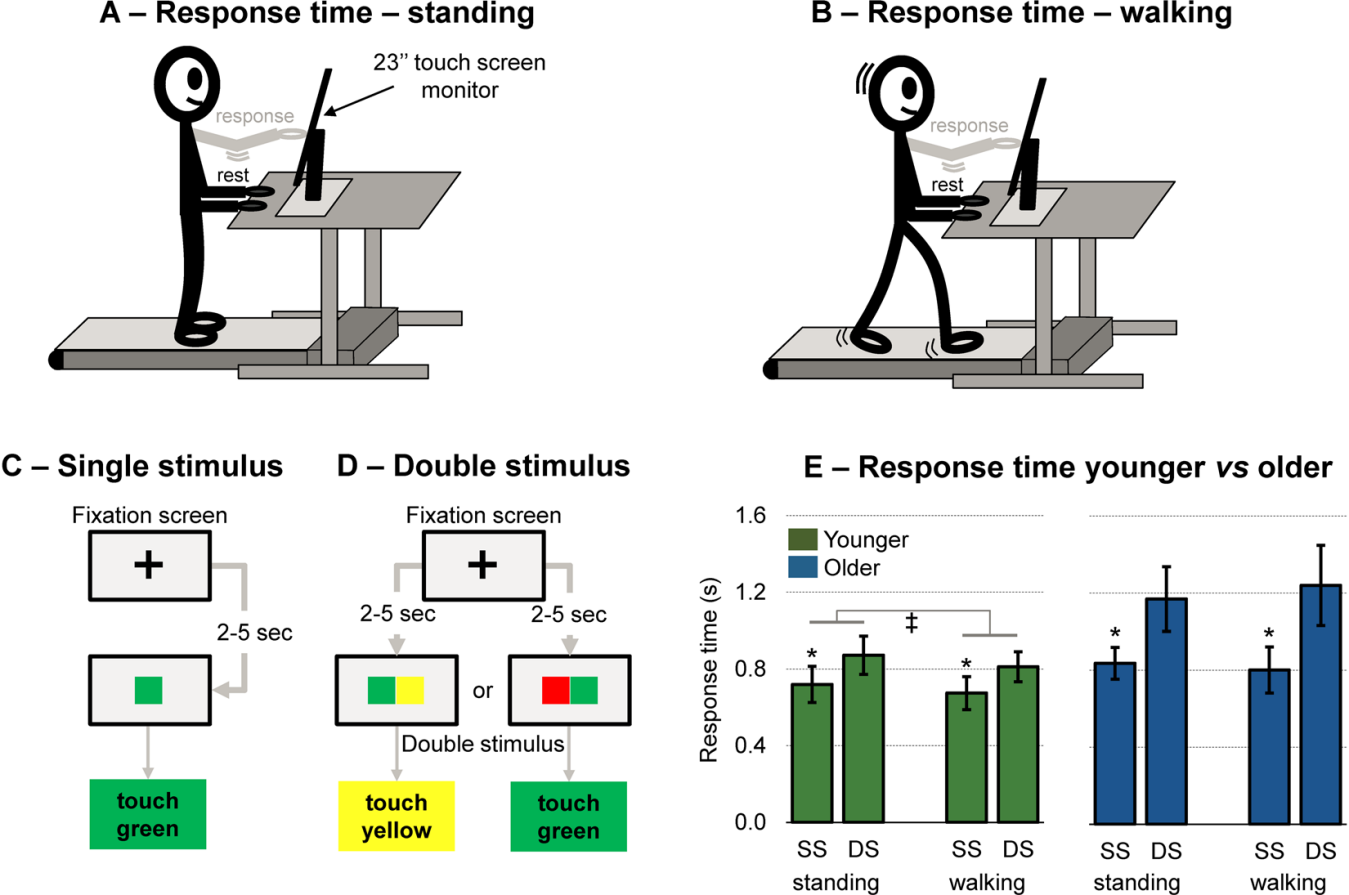
Experimental protocol to test cognitive-motor interference in different postures. Participants were asked to stand (panel A) or walk at preferred speed (panel B) with their hands resting on a desk. A touch-screen monitor was placed on the desk at a reachable arm distance. A computer-controlled cognitive paradigm involved the appearance of a fixation cross in the middle of the screen from 2 to 5 seconds intervals (randomly selected). In the single stimulus condition (SS, panel C) a single green square appeared replacing the fixation cross, and participants should touch inside the area of the square as fast as possible with their dominant hand. In the double stimulus condition (DS, panel D), a pair of green + yellow or red + green squares appeared replacing the fixation cross. If the pair of squares were green + yellow, participants should touch inside the yellow square. If the pair of squares were red + green, participants should touch inside the green square. In the panel E, mean ± SD response time for the standing single stimuli, standing double stimuli, walking single stimuli and walking double stimuli. ^‡^Denotes significant difference in relation to walking condition in the same group (p < 0.005); *denotes significant difference in relation to double stimulus condition in the same group (p < 0.0001).

The response time protocol comprised a standing task (Fig. 2A), and a walking task (Fig. 2B). In each type of posture, the participants reacted as fast as possible to the appearance of a single stimulus (Fig. 2C) or a double stimulus (Fig. 2D) on a touch screen. The response time for both groups are shown in Fig. 2E. When the response time of both groups were considered in a 2-way ANOVA with repeated measures, we found a significant interaction among posture (standing *vs* walking) × stimuli (single *vs* double stimulus) × group (younger *vs* older adults) (F(2,33) = 7.81; p < 0.01; ŋp^2^ = 0.187). Post-hoc analysis revealed that older adults have significantly slower response time when double stimuli were presented during walking when compared to younger participants in both standing and walking tasks (p < 0.001).

When the response time was analyzed for each group separately, only main effects of posture (F(1,17) = 15.36, p < 0.005, ŋp^2^ = 0.475) and stimuli (F(1,17) = 52.52, p < 0.0001, ŋp^2^ = 0.755) were found for the younger participants, which presented 22 ± 16% slower response time for the double stimulus condition compared to single stimuli and during walking compared to standing. For the older adults group, it was found a significant posture × stimuli interaction (F(1,17) = 7.58, p < 0.05, ŋp^2^ = 0.308). Further post-hoc tests showed that the older adults were 40 ± 24% slower to respond to double stimuli during walking when compared to single stimuli during standing (p < 0.0001) and walking (p < 0.00001). However, no difference between standing and walking was found in the double stimulus condition (p > 0.05). The different results between groups demonstrate specific effects depending on the group.

### Overground walking speed

**Figure 3:**
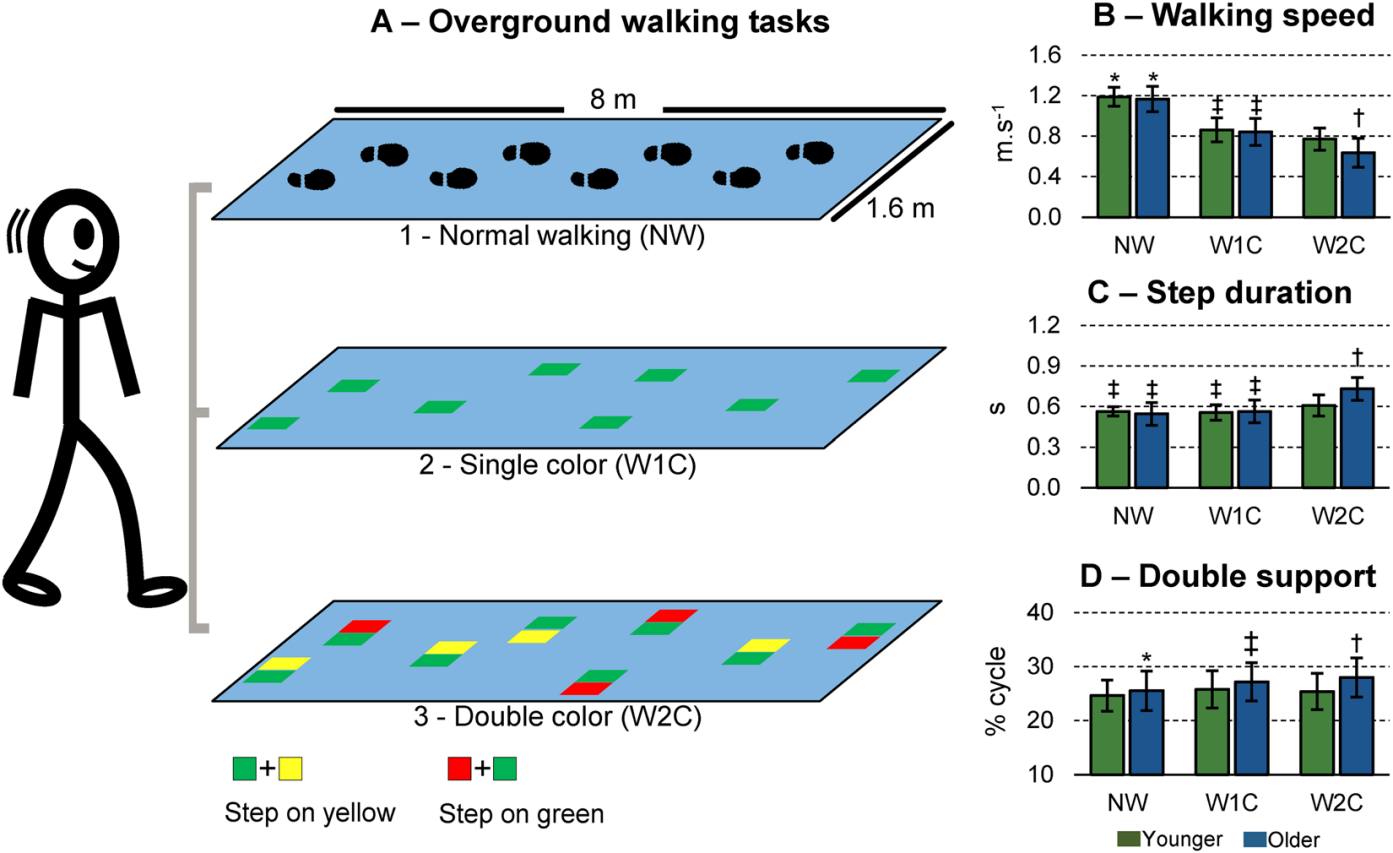
Overground walking protocol (**A**). Younger and older adults walked at preferred speed (1, NW), at preferred speed while following pre-established clues on the floor (2, W1C) and at preferred speed while having to step only on the correct color (3, W2C). Participants should step on the yellow clue if the pair of clues was green + yellow, whereas they should step on the green clue if the pair of clues was red + green. The mean ± SD walking speed (**B**), step duration (**C**) and double support period (**D**) were calculated for each condition. *Denotes significant difference in relation to W1C and W2C (p < 0.05); ^‡^Denotes significant difference in relation to W2C (p < 0.05); ^†^Denotes significant group × condition interaction, in which older adults were different in relation to the younger group in W2C (p < 0.05).

Younger and older adults walked overground in three different conditions: (1) normal walking at preferred speed (NW); (2) while following a pre-established path using green clues marked on a mat (W1C) and (3) following pairs of clues marked on the floor, in which they had to remember which color was allowed to step onto (W2C, Fig. 3A). There was a significant condition × group interaction for the walking speed (F(2,33) = 6.45, p < 0.005, ŋp^2^ = 0.281, Fig. 3B). Post-hoc analysis revealed faster walking speed for NW in comparison to W1C (p < 0.0001) and W2C (p < 0.0001) for both groups. In addition, W1C presented faster walking speed in comparison to W2C for both groups (p < 0.0001). Moreover, the older adults group presented significantly slower W2C walking speed in comparison to the younger adults (p < 0.005).

### Step duration

There was a significant condition × group interaction for the step duration (F(2,33) = 15.64, p < 0.0001, ŋp^2^ = 0.487, Fig. 3C). Post-hoc analysis revealed significantly longer step duration for W2C in comparison to NW and W1C for the younger group (p < 0.05) and especially older adults group (p < 0.0001). Moreover, the older adults group presented significantly longer W2C step duration in comparison to the younger adults (p < 0.0001).

### Double support

There was a significant condition × group interaction for the step duration (F(2,33) = 3.50, p < 0.05, ŋp^2^ = 0.175, Fig. 3D). Post-hoc analysis revealed shorter double support duration for NW in comparison to W1C (p < 0.05) and W2C (p < 0.005), as well as between W1C in comparison to W2C (p < 0.05) only for the older adults group. Moreover, the older adults group presented significantly longer double support period during W2C in comparison to the younger adults (p < 0.05).

### Vertical force and center of pressure speed

**Figure 4:**
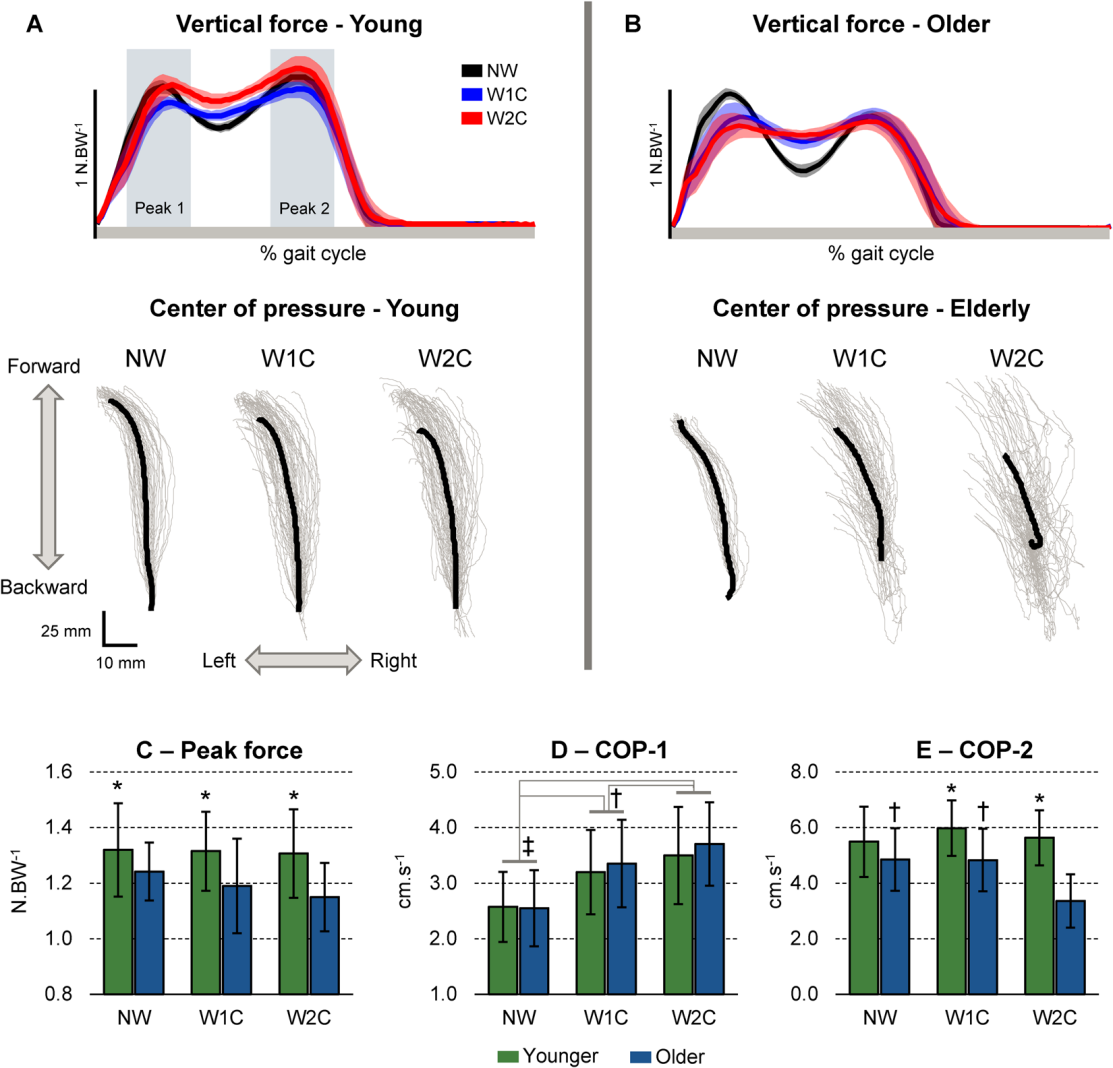
Illustrative right side vertical ground reaction force and center of pressure trajectory from one younger (**A**) and one older adult (**B**) walking at preferred speed (NW, *black*), at preferred speed while following pre-established clues on the floor (W1C, *blue*) and at preferred speed while having to step only on the correct color (W2C, *red*). The two vertical shaded areas in A represent a period of ~100 ms around the first (Peak 1) and second (Peak 2) vertical peak forces. Thick lines represent mean force and shaded area represents ± 1 standard deviation, while the gray traces in the center of pressure plots represent individual steps. The mean ± SD peak vertical force (**C**), medial-lateral center of pressure speed 100 ms around the first vertical peak force (COP-1, **D**) and medial-lateral center of pressure speed 100 ms around the second vertical peak force (COP-2, E) were computed for each condition. *Denotes significant difference in relation to older adults (p < 0.005); ^‡^Denotes significant difference in relation to W1C and W2C (p < 0.005). ^†^Denotes significant difference in relation to W2C (p < 0.005).

While younger participants presented similar patterns for vertical ground reaction forces and center of pressure across conditions (Fig. 4A), older adults changed their walking towards reducing peak loading, and highly variable center of pressure trajectories in W1C and especially W2C (Fig. 4B). There was a main effect of group in the first peak of the vertical force (i.e., the braking force at initial contact) during walking (F(2,33) = 9.43, p < 0.005, ŋp^2^ = 0.217, Fig. 4C), in which the older adults group presented lower peak force when compared to the younger group. For the medial-lateral center of pressure speed 100 ms around the first vertical peak force (COP-1), there was a significant main effect of condition (F(2,33) = 23.49, p < 0.0001, ŋp^2^ = 0.587, Fig. 4D). Post-hoc test revealed that the COP-1 during NW was lower in comparison to W1C and W2C for both groups (p < 0.005), and that COP-1 during W1C was lower in comparison to W2C for both groups (p < 0.05).

Regarding the medial-lateral center of pressure speed 100 ms around the second vertical peak force (COP-2), there was a significant condition × group interaction (F(2,33) = 6.31, p < 0.005, ŋp^2^ = 0.203, Fig. 4E). Post-hoc analysis revealed that COP-2 during W2C was lower in comparison to NW and W1C only for the older adults group (p < 0.0001). Moreover, the older adults group presented longer double COP-2 for W1C (p < 0.005) and W2C (p < 0.0001) in comparison to the younger adults.

### Relationship between overground walking speed vs response time

**Figure 5:**
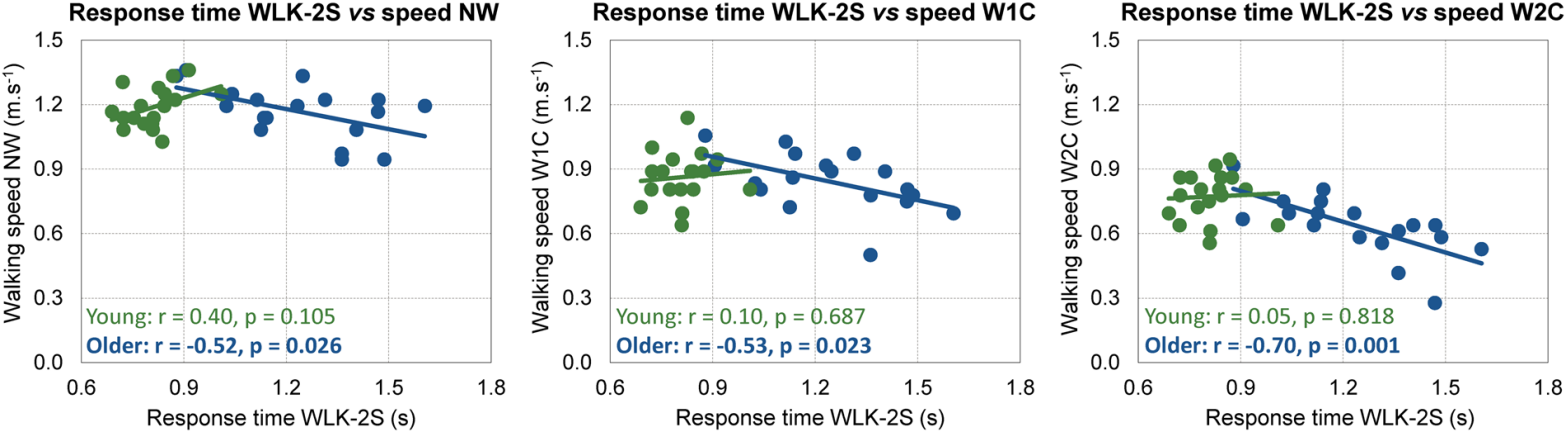
Pearson correlation coefficient different response time conditions (*x axis*) and overground walking speed (*y axis*) for younger (*green dots and trend lines*) and older adults (*blue dots and trend lines*). The response time to double stimuli during walking (WLK-2S, *lower row*) was correlated to the walking speed during walking at preferred speed (NW, *left*), at preferred speed while following pre-established clues on the floor (W1C, *middle*) and at preferred speed while having to step only on the correct color (W2C, *right*). Pearson correlation coefficient (r) and significance level (p) are shown for each comparison.

The Pearson correlations between walking speed and response time during NW, W1C and W2C were significantly different between younger and older adults (p < 0.05). Younger participants presented no significant correlations between the walking speed in the NW, W1C or W2C (p > 0.05, Fig. 5) with respect to the response time in the double stimulus condition during walking. On the other hand, walking speed for older adults presented moderate inverse correlations with the response time to double stimuli during walking for NW (Fig. 5, *left panel*) and W1C (Fig. 5, *middle panel*). There was an especially strong inverse correlation for W2C walking speed (r = −0.7, p < 0.001, Fig. 5, *right panel*).

## Discussion

In this study, two protocols were developed where the difficulty to complete an upper-limb reaching task and a locomotor task were manipulated by increasing the cognitive complexity using distractors. Reductions in motor performance would be expected for both younger and older adults by increasing the cognitive complexity of the tasks, regardless of their physical fitness levels. However, if performing general physical activities could preserve cognitive functions in older adults, we hypothesized that there would be no differences between the deficits presented by younger and older physically active adults. Our results demonstrated that older adults presented a markedly reduced motor performance when they were challenged by a complex decision-making task on both upper limb and precision stepping walking tasks. In addition, we further explored possible correlations and found a strong inverse correlation between walking speed (overground walking) and response time to double stimuli (during treadmill walking) during complex decision-making tasks. It was observed that older adults presenting the lowest walking speed were also those presenting the longest response times when double stimuli were presented. These findings suggest that cognitive deficits caused by age may globally influence motor control.

The results from the postural sway and normal overground walking tests suggest similar performance between active younger and older adults. This is in line with other studies which presented similar walking speeds^36^ and postural stability^26^ when comparing physically active older and younger adults. These results reinforce the positive impact of physical activities in static postural stability and mobility for older adults. Despite these similarities, older adults produced lower braking forces during the first peak vertical forces regardless whether the walking test presented cognitive context. This reduced braking force is related to motor adaptations older adults undergo to reduce force absorption in the braking phase of stance^32^.

The older adults in this study presented an overall longer response time when compared to younger participants. More importantly, these older adults presented a greater influence of task complexity on response time. With respect to the overground walking task, older adults presented similar gait kinematics (walking speed, step duration and double support period) compared to younger adults during simple walking task and also when following only one color (W1C). However, including a decision-making context during the precision stepping task remarkably reduced the performance of older adults. Therefore, older adults presented reduced performance when decision-making was part of the task, regardless if it was an upper limb reaction time context or a precision stepping context.

Ageing has been associated with reductions in the performance of reaction time tasks^3,21,39,40^, as well as poorer motor performance during precision stepping tasks^16,41^. Deficits in executive function, working memory and reduced brain mass, especially at the frontal lobe^7,17^, may explain the poorer performance of the older adults to perform the decision-making part of these different motor tasks. Moreover, degeneration of cerebellum and the proprioceptive system may contribute to slowing multi-joint movement^17^, which in this case may affect predominantly the response time task, but may influence walking performance as well. A recent study with similar overground walking experimental design has shown increased participation of the frontal area of the brain during the decision-making context in young adults^42^. We speculate that older adults may present similar trends of increased participation of brain centers related to decision-making and cognition.

The ability to react promptly to various stimuli has been considered a relevant falls predictor^3,20^, and our results contribute to this concept by suggesting that response time, in physically active older adults, can be substantially influenced by cognitive demands. Similarly, our results demonstrated that older adults presented reduced walking speed, increased step duration and periods of double support, coupled with the reductions in the medial-lateral center of pressure speed during precision stepping involving decision making. As a coping mechanism for walking amid dual task challenges, young adults reduce their walking speed, whereas older adults seem to reduce both walking speed and swing time^10^, as well as increase double support periods^30,31^. Moreover, minimizing medial-lateral changes in foot position at the end of double support phase may help older adults to be confident in taking a further step in such challenging conditions.

Younger adults presented faster response time during treadmill walking when compared to the standing, regardless of the complexity of the task, which contrasts previous literature^7,30^. Rhythmic and discrete movements require the activation of similar brain areas, but discrete movements may require the activation of additional cortical planning areas^7^. On the other hand, performing a response time task during walking is a combination of a highly encoded rhythmic task and discrete motor tasks, and this combination involves networks with lower influence on gait control^43,44^. Therefore, there may be a lower demand for sharing brain resources, explaining the maintenance of response times during walking especially for the younger group. Moreover, the act of walking may increase the readiness of brain areas related to movement^45,46^, optimizing prompt reactions to stimuli. Similarly, the lack of differences between the standing and walking response time for the older group may be related to such increased brain readiness that could compensate cognitive declines. Moreover, the treadmill walking modulation can be predominantly ruled by spinal mechanisms such as central pattern generators, as well as the cerebellum^47,48^. These both modulating mechanisms are not susceptible to interferences caused by cognitive control, which may explain the performance maintenance during the walking task. To our knowledge, no studies have focused directly on this particular phenomenon. Therefore, further studies are needed to elucidate this controversy.

Performing upper limb movements and locomotion are two different motor activities, and we hypothesized that including a cognitively demanding context in both tasks would compromise the motor performance in older adults. Moreover, we hypothesized that the magnitude of such influence would be similar across tasks, and this hypothesis was confirmed by the strong inverse correlation between walking speed (overground walking) during complex decision-making tasks and response time to double stimuli (during treadmill walking). The correlation is inverse because both higher walking speed and shorter response time are indicators of better performance. It was evident that older participants with lower walking speed presented the longest response times. These findings suggest that cognitive deficits caused by age may globally influence motor control, not only one type of motor ability. Future studies are needed to further explore this evidence, by conducting multiple types of motor-cognitive tasks in the same older participants and describe their relative deficits across these tasks.

Recent studies have highlighted the importance of including cognitive tasks when designing exercise training programs prevent falls in older adults^23,24,38^. In practice, our findings demonstrating reduced motor performance in physically active older adults are highly relevant for health professionals, as these findings support the need for designing exercise training programs involving a combination of motor abilities and cognitive function. Moreover, the protocols proposed in this study could be implemented in future research intervention protocols dedicated to improving executive functions in older adults. Further, tests using the principles of the presented cognitive-motor tasks could potentially benefit middle-aged people, as a screening test for fall incidents.

It is noteworthy that if physical activities can help preserving cognitive function with age^38^, it can be speculated that sedentary older adults would present even greater limitations to perform the protocols explored in this study. However, this study did not investigate sedentary younger and older adults, which is a limitation that narrows the relevance of our results only to physically active individuals. Therefore, a future study comparing sedentary younger and older adults will contribute to assess the investigated influences of cognition on movement control in physically inactive people. Such study can increase our understanding on the role of physical activities on motor tasks involving cognitive demands.

In summary, this study found that both young and older adults are susceptible to reductions in response time and precision stepping performance if these tasks include complex decision-making. However, the decrement in performance was greater in both tasks for the older adults. In addition, older adults walked slower, with longer periods in double support and reduced medio-lateral center of pressure speed at the end of stance when walking was guided by a decision-making context. It is noteworthy that the poorer performance of older adults during precision stepping was only present when the task included decision making. Moreover, older adults most affected by decision-making presented compromised performance in both tasks, suggesting a global effect of cognitive demands on motor control. Therefore, performing physical activities regularly can help older adults maintaining levels of postural control comparable to younger adults in basic postural tasks. However, physically active lifestyle does not assure comparable motor performance when decision-making is required.

## Methods

### Participants

Thirty-six clinically healthy participants, 18 younger adults and 18 older adults participated in this study. The physical activities practiced by young adults were: team sports (handball, football, volleyball), running, cycling, racket sports (tennis, badminton) and resistance training. The physical activities practiced by older adults were: team sports (volleyball, football), walking/running, cycling, racket sports (tennis, badminton), group classes (aerobic exercises, stretching) and resistance training. Table 1 shows the demographic characteristics of the two groups, as well as the weekly training volume in different physical activity intensities. Exclusion criteria for this study were: visual and walking impairments, vestibular dysfunctions, a history of lower back and/or lower-extremities pain and/or injuries in the past 6 months and a shoe size smaller than 37 (EU). In addition, participants should not have engaged in any type of cognitive training activities such as Lumosity, Fit Brains Trainer, Cognito and others for the past 6 months. The footwear was selected from their own personal sports shoes following certain attributes like: tight laced, low cut shoes and heel raise between 0–15 mm. Written informed consent was obtained from all the participants prior to testing. The study was approved (N-20160042) by The North Denmark Region Committee on Health Research Ethics and all methods conformed to the standards of the Declaration of Helsinki.

### Physical activity

Physical activity level was assessed using the short version of the International Physical Activity Questionnaire (IPAQ), which is proven a national and regional valid and reliable assessment of physical activity level^49^. The original questionnaire was offered in Danish and English. Participants reported the amount of time practicing mild, moderate and vigorous physical activities in the past 7 days, and the interpretation of what is a mild, moderate or vigorous activity was based on their own perception. One person in each group was classified as “low” level of physical activity. The participant in the older group reported 100 minutes of physical activities (40 min in vigorous activities), and the young participant reported 80 minutes of physical activities (50 of which were moderate for that particular week.

### Experimental protocol

In a single session, participants performed cognitive and postural tasks divided into three blocks: (1) simple postural tasks, which consisted of postural sway and normal overground walking; 2) overground walking with precision stepping following single or double stimuli; and 3) assessment of the response time to a single or double stimulus on a touch screen monitor while standing and walking on a treadmill. The postural sway and overground walking were fixed as the first block in the session, whereas the order for the second and third blocks were randomized. Participants wore an in-shoe plantar system recorder to collect plantar pressure.

### Postural sway

Participants were asked to stand as still as possible for 30 seconds with the feet 10 cm apart at a 1 m distance from a white wall. A reference black dot was positioned on the wall, in the participant sight height, as the fixation point throughout the recording. Plantar pressure was recorded from three trials.

### Overground walking

Participants were asked to walk at their preferred speed through an 8 m long and 1.6 m wide solid dark blue walkway. For each trial, participants were instructed to walk through the mat and decelerate only after leaving the mat, stop and turn, and wait for the experimenter’s signal to perform the next trial. The experiment consisted of three different walking tasks: (1) normal walking at preferred speed (NW, Fig. 2A, *top row*), (2) walking on a pre-defined pathway in which 16 green 10 × 12 cm clues on the blue mat defined stepping position and imposed variation in step width and length (W1C, Fig. 2B, *center row*), and (3) walking on the same pre-defined W1C pathway, however the place for each step would vary depending on the combination of three different colors (green, yellow and red) placed side-by-side (W2C, Fig. 2C, *bottom row*).

For both W1C and W2C tasks, participants were asked to walk through half of the walkway twice for familiarization purposes. In W2C, for each step the participant had to follow these rules: if green & yellow clues: then step on yellow; if green & red clues: then step on green. These rules added a cognitive decision process between each step. Participants were instructed to keep walking and avoid stopping while thinking about the next step, as well as to keep walking if they mistakenly stepped onto incorrect color marks. Walking trials in which participants stopped walking were discarded. A total of 10 walking trials through the mat were performed while walking speed and plantar pressure were recorded. For all tasks, an auxiliary researcher was recording the walking speed disregarding the transition zones for every trial. The same researcher also registered the number of mistakes made by the participants during W2C.

### Response time

Participants were asked to either stand or walk on a treadmill desk (Lifespan TR1200B, Salt Lake City, USA), in which a touch screen monitor (HP Elitedisplay E230T 23”, response time: 5 ms, refresh rate: 60 Hz) was fixed for the visual stimulus presentation. The 5 ms refresh rate was accounted for in the results section. The height of the desk was adjusted to accommodate the participant’s hands with the elbows flexed at 90 degrees. Participants performed the response time task while standing (Fig. 3A) and while walking at the preferred speed computed from the NW condition (Fig. 3B). The task consisted of reacting to the appearance of stimulus presented on the touch screen, by tapping in specific colored rectangles. The stimulus presentation and response time recording were conducted through a custom MATLAB^®^ script (R2016b, Mathworks Inc., Natick, MA USA).

Two stimulus types were tested: (1) single stimuli (Fig. 3C), in which participants should react to a single 6 × 8 cm rectangle consistently appearing in the center of the screen; and (2) double stimuli (Fig. 3D), in which participants should react to the appearance of two 6 × 8 cm rectangles of different colors appearing side-by-side in the center of the screen. Similarly, to the W2C task, participants had to follow these rules: if green & yellow clues: touch the yellow rectangle, if green & red clues: touch the green rectangle. For each stimulus presentation, a fixation cross was presented in the center of the screen, indicating that the participant should focus and be ready to react. The fixation cross was replaced by the stimulus after a random period between 2 and 5 seconds, and participants were instructed to touch the target stimulus as fast as possible. If the participant touched the wrong rectangle, the attempt was marked as a mistake.

Participants were allowed to familiarize to the single and double stimuli conditions prior to the data collection. For each condition, 10 responses were recorded, and the five fastest response times were averaged to represent the participant’s response time. It was possible to use five trials as there was a low inter-trial variability across the five fastest response times for both young (4.75 ± 2.88%, 95% CI = 4.07–5.42%) and older adults (6.79 ± 4.13%, 95% CI = 5.81–7.75%, more details in the supplementary material *Response time – Inter-trial variability*). If a mistaken trial was within the five fastest, it would be also accounted in the average, but no mistaken trials were marked within the five fastest for any participant in this experiment. The order of the tasks (standing *vs* walking) and the order of the conditions (single *vs* double stimuli) were randomized for each subject. In addition, the positioning of the color order of the rectangles in the double stimulus condition was randomized. A preliminary study in an independent sample of 16 physically active younger adults (30 ± 4.4 years) was conducted to establish the test-retest reliability using intra-class correlation coefficient. The test-retest reliability was good (0.88) for the single stimuli (in both standing and walking conditions) and medium (0.68) for the double stimuli (in both standing and walking condition).

### Data analysis - Postural sway

Bilateral vertical force, medial-lateral and anterior-posterior center of pressure data were recorded using a plantar pressure system (Pedar-X Recorder, Novel GmbH, Munich, Germany) at 100 Hz sampling frequency. The plantar pressure insoles were placed in the participants’ shoes in a manner that all the 99 resistive sensors did not present creep by bending. Prior to analysis, force and center of pressure data were low-pass filtered (50 Hz, zero-lag butterworth 4^th^ order). The average center of pressure speed and the variability of the center of pressure speed (calculated as the standard deviation of the center of pressure speed) were computed from the 30 s plantar pressure standing data^50^ and were analyzed using custom scripts on MATLAB^®^ (R2016b, Mathworks Inc., Natick, MA USA).

### Data analysis – Overground walking

The walking speed was defined as the time between the first step on the mat and the first step out of the mat. The vertical force from the right side was used to segment individual gait cycles. Foot contact to the floor was defined when the raw vertical force exceeded 20 N. The following gait parameters were extracted from the vertical force curves: (1) peak force: defined as the peak vertical force within the first 40% gait cycle, normalized by body weight; (2) step duration: defined as the time between the right initial contact to the subsequent left initial contact to the ground; (3) double support duration: defined as the period in which both feet are in contact with the mat. The double support duration was normalized in relation to the stride duration. In addition, the first and second vertical peak force instants were defined for each gait cycle, and the medial-lateral center of pressure speed was computed by deriving the pressure position vectors in a 100 ms window around these peaks *(see top parts of* Fig. 4A and B *for illustration*).

### Statistical analysis

Statistical analyses were performed using the Statistical Package for Social Sciences (SPSS Version 24, IBM Corporation, Armonk, New York, USA) software. The normality of the dependent variables was confirmed using Shapiro-Wilk test. Between-group differences in age, height, weight and physical activity levels were assessed using independent t-tests. For the response time analysis, the within-subject effects of stimuli (single stimuli *vs* double stimuli), posture (standing *vs* walking) and the between-subject effects of group (younger *vs* older adults) were assessed by a 2-way repeated measure ANOVA with mixed factors. As a significant posture × stimuli × group interaction was found, a post-hoc pairwise comparison was conducted in the two groups separately. For the other dependent variables (walking speed, step duration, double support duration, peak force and medial-lateral center of pressure speed), the within-subject effects of condition (NW *vs* W1C *vs* W2C) and the between-subject effects of age (younger *vs* older adults) were assessed by a 2-way repeated measure ANOVA with mixed factors. Bonferroni pairwise post-hoc tests were used in case of significant condition × group interaction. Partial eta-squared (ŋp^2^) was used to calculate the effect sizes of the statistical results. The relationship between the walking speed in the three different overground walking tasks (NW, W1C and W2C) and the response time recorded in the double stimulus condition while walking was investigated using Pearson correlations. The the effect of age (young vs older adults) on the correlation coefficients was assessed by the method described in Fisher^51^. Subsequently, the correlation coefficients were defined as very weak (0 to 0.19), weak (0.2 to 0.39), moderate (0.4 to 0.59), strong (0.60 to 0.79) and very strong (0.8 to 1)^52^. All dependent variables demonstrated a normal distribution and the average statistical power ranged from 0.83–0.92. The significance level was set at p < 0.05. Data are displayed as mean ± standard deviation (SD).

## Electronic supplementary material

Supplementary Analysis
