# Supplementary material for: The cognitive complexity of concurrent cognitive-motor tasks reveals age-related deficits in motor performance
Source: Sci Rep. 2018 Apr 17;8:6094. doi: 10.1038/s41598-018-24346-7 (PMC5904104; doi:10.1038/s41598-018-24346-7)
Supplement: Supplementary file 1 — Supplementary Analysis [file 41598_2018_24346_MOESM1_ESM.docx]

**THE COGNITIVE COMPLEXITY OF CONCURRENT COGNITIVE-MOTOR TASKS REVEALS AGE-RELATED DEFICITS IN MOTOR PERFORMANCE**

**Authors**

Anderson Souza Oliveira^1^

Mikkel Staall Reiche^2^

Cristina Ioana Vinescu^2^

Sif Amalie Halkjær Thisted^2^

Carina Hedberg^2^

Miguel Nobre Castro^1^

Martin Gronbech Jørgensen^3^

**Affiliations**

1 – Department of Materials and Production, Aalborg University, Aalborg – Denmark

2 – Department of Health Science and Technology, Aalborg University, Aalborg – Denmark

3 – Department of Geriatrics, Aalborg University Hospital, Aalborg – Denmark

**Supplementary analysis - Inter-trial variability of the response time**

The response time data registered from both the younger and older adults groups were used in this analysis. For each participant, the average of the 5 fastest response times (out of 10) were used to describe the results in each condition. This analysis shows the inter-subject variability based on the coefficient of variation (CV) for each group and condition. Therefore, this analysis described how variable were these five trials. The CV is calculated as the ration of the standard deviation to the mean of the five trials. In Figure 1, we show the CV for the younger group (top plot) and for the older group (bottom plot)

**Figure 1**. Boxplot representing the coefficient of variation (CV) across the 5 fastest trials recorded during each of the experiment conditions: single stimulus during standing (1-STD), single stimulus during walking (1-WLK), double stimulus during standing (2-STD) double stimulus during walking (2-WLK). In each plot the central red mark is the median; the edges of the box are the 25th and 75th percentiles. The whiskers cover approximately 99% of the data. + denotes CV values outside the box limits.

It was possible to use five trials as there was a low inter-trial variability across the five fastest response times for both young (4.75±2.88%, 95% CI = 4.07-5.42%) and older adults (6.79±4.13%, 95% CI = 5.81-7.75%,
